# Number of COVID-19 patients classified as cured: an imminent danger for the population

**DOI:** 10.31744/einstein_journal/2020CE6146

**Published:** 2020-10-15

**Authors:** Marcos Roberto Tovani-Palone, Stefano Lacagnina, Lorenzo Ferro Desideri

**Affiliations:** 1 Universidade de São Paulo Faculdade de Medicina de Ribeirão Preto Ribeirão Preto SP Brazil Faculdade de Medicina de Ribeirão Preto, Universidade de São Paulo, Ribeirão Preto, SP, Brazil.; 2 Università degli Studi Palermo Italy Università degli Studi di Palermo, Palermo, Italy.; 3 University of Genoa Genoa Italy University of Genoa, Genoa, Italy.

Dear Editor,

Several studies have been carried out and highlighted the severity of many cases of the coronavirus disease 2019 (COVID-19).^(^^1^^–^^3^^)^ Nonetheless, the criteria that have been adopted so far by many countries, such as Brazil, for progression to cure of patients with mild symptoms or asymptomatic individuals, may pose risks due to some limitations in monitoring the evolution of the infection. In such situations, the current recommendation includes laboratory tests to confirm cure only in the severe cases of the disease. For asymptomatic patients or those with mild symptoms, even with laboratory confirmation for the initial diagnosis, most will follow only the clinical criteria to establish a final classification of cure, which is generally based on reports of patients.^(^^4^^)^ Considering the possibility of intermittent or persistent symptoms in patients who had mild symptoms for over 14 days,^(^^5^^)^ and the impracticability of providing appropriate clinical criteria to assess the asymptomatic cases, the number of cured individuals may be considerably overestimated worldwide. In addition, asymptomatic individuals who are not cured yet may resume their work and routine activities, placing a large proportion of the population at risk.
