# Supplementary material for: Number of COVID-19 patients classified as cured: an imminent danger for the population
Source: Einstein (Sao Paulo). 2020 Oct 15;18:eCE6146. doi: 10.31744/einstein_journal/2020CE6146 (PMC9586428; doi:10.31744/einstein_journal/2020CE6146)
Supplement: Translation

# Translation: Número de pacientes classificados como curados da COVID-19: um perigo iminente para a população

**Authors:** Marcos Roberto Tovani-Palone, Stefano Lacagnina, Lorenzo Ferro Desideri

**Affiliations:** 1 Ribeirão Preto SP Brasil Faculdade de Medicina de Ribeirão Preto, Universidade de São Paulo, Ribeirão Preto, SP, Brasil.; 2 Palermo Italy Università degli Studi di Palermo, Palermo, Italy.; 3 Genoa Italy University of Genoa, Genoa, Italy.

Caro Editor,

Diversos estudos têm sido conduzidos destacando a gravidade de muitos casos da doença pelo coronavírus 2019 (COVID-19).^(^^1^^–^^3^^)^ Contudo, os critérios adotados até então por muitos países, como o Brasil, com respeito à evolução para cura de pacientes assintomáticos ou com sintomas leves, podem representar riscos, devido a algumas limitações para o monitoramento da evolução da infecção. A recomendação atual, nesses casos, prevê a realização de testes laboratoriais confirmatórios de cura apenas para os casos graves da doença. Os casos de pacientes com sintomas leves ou assintomáticos, mesmo com confirmação laboratorial para o diagnóstico inicial, seguem, na maior parte das vezes, apenas critérios clínicos para estabelecimento de classificação final como cura, com base geralmente em relatos dos pacientes.^(^^4^^)^ Diante da possibilidade de persistência dos sintomas de forma intermitente em pacientes com sintomas leves por período superior a 14 dias^(^^5^^)^ e da inviabilidade de prover critérios clínicos adequados para avaliação de casos assintomáticos, o número de curados no mundo pode estar consideravelmente superestimado. Além disso, pessoas assintomáticas ainda não curadas podem estar retornando aos seus postos de trabalho e às atividades de rotina, colocando em perigo grande parte da população.
